# A Comparison Analysis Between Pre-departure and Transitioned Expat-Preneurs

**DOI:** 10.3389/fpsyg.2020.588169

**Published:** 2021-01-08

**Authors:** Vilmantė Kumpikaitė-Valiūnienė, Jurga Duobienė, Antonio Mihi-Ramirez

**Affiliations:** ^1^Digitalisation Research Group, Kaunas University of Technology, Kaunas, Lithuania; ^2^Department of International and Spain’ Economics, University of Granada, Granada, Spain

**Keywords:** entrepreneurship, expatriate entrepreneurs, expat-preneurs, pre-departure expat-preneurs, transitioned expat-preneurs, self-initiated expatriates

## Abstract

This paper contributes to the understanding on the reasons that lead to entrepreneurship in other countries. We focus on expat-preneurs, those who decided to undertake business opportunities in other countries (before or after settling there). Using comparison analysis and logistic regression, we examine pre-departure and transitioned expat-preneurs’ demographic characteristics and push-pull factors that lead them to expatriate. From a survey conducted in 2015-2016 of 5,532 Lithuanians expatriated in 24 countries, a sample of 308 respondents with their own businesses abroad was selected. This research contributes to the literature on expat-preneurs, with empirical evidence on pre-departure and transitioned self-initiated (SI) expat-preneurs. The results revealed that demographic features matter when studying such global entrepreneurs. It is a process experienced differently by males and females and, as such, it can be considered as gender selective. Thus, more pre-departure expat-preneurs are male than female, but there is a growing number of female transitioned expat-preneurs. Pre-departure expat-preneurs are older and less educated than transitioned ones and have been pushed to move abroad by issues such as political corruption or a non-supportive tax system, and are attracted by a higher possibility of self-realisation as well as the prestige of the host country. Meanwhile, transitioned expat-preneurs have been pushed to emigrate due to family reasons or too few employment opportunities in their home country.

## Introduction

Nowadays, more and more people work abroad. In 2017, it was estimated that there were 66.2 million expatriates worldwide, which represents 0.77 percent of the total global population (Finaccord, 2018; Hussain et al., 2019). “Being rooted in a profession rather than a country and trying to find the best possibility to work in that profession without being limited by national borders is what reflects the reality of many – especially highly skilled – individuals of our time” (Agha-Alikhani, 2018, p. 2).

The growing involvement of expatriates in the development of entrepreneurial businesses has been observed together with the increasing expatriation numbers (Sekliuckienė et al., 2014; van Rooij and Margaryan, 2019; Internations, 2020). Moreover, Baycan-Levent and Nijkamp (2009) highlighted that, in general, foreigners are more likely to become entrepreneurs than similarly skilled native-born workers, and self-employment rates of foreigners in many countries exceed those of native-born.

Entrepreneurship of foreigners in host countries is a traditional field of interest for scholars who analyse diaspora entrepreneurship (Vemuri, 2014; Elo, 2016), migrant entrepreneurs (Engelen, 2002; Aliaga-Isla and Rialp, 2013; Sahin et al., 2014; van Rooij and Margaryan, 2019), and expatriate entrepreneurs (Du Plessis, 2009; Connelly, 2010; Zgheib and Kowatly, 2011). Despite these mentioned concepts, Vance et al. (2016) and, later, Selmer et al. (2018) proposed a new meaning of self-employed expatriates: expat-preneurs. These are not entrepreneurs within the context of “South to North” migration (a.k.a. “ethnic entrepreneurs” or “immigrant entrepreneurs”) but are a new and growing reality of foreign global entrepreneurs who come from developed economies (Girling and Bamwenda, 2018), a definition which entails several differences, advantages, and disadvantages over traditional “ethnic entrepreneurs” (Selmer et al., 2018; van Rooij and Margaryan, 2019).

To date, expat-preneurs by themselves are not a very much analysed phenomena, despite the current context of globalisation. Vance et al. (2016) presented a concept of expat-preneurs, dividing them into pre-departure and transnational expat-preneurs, and posed potential research questions in this field. Paik et al. (2017) theoretically analysed self-initiated expatriates (SIEs) who become expat-preneurs and Selmer et al. (2018) focused on a comparison of SIEs with expat-preneurs coming from assigned expatriates (AEs). However, the aim of this paper is to compare the demographic characteristics and motivations to expatriate of pre-departure and transnational SI expat-preneurs, something that has not been done in previous studies.

As the basis for this study, we concentrate only on Lithuania. Since the restitution of Lithuanian independence in 1990 and the collapse of the Soviet Union, the Lithuanian net migration indicator has been negative (Migration in numbers, 2020). Therefore, Lithuania is a good example for a deeper look at the phenomena of expatriation. The following comparison analysis is based on Lithuanian expat-preneurs (people who moved from Lithuania and established businesses abroad).

Our paper is organised as follows. First, the meaning of expat-preneur is presented, with the focus on two types in particular: pre-departure and transitioned expat-preneurs. Second, the concept of expat-preneur and its demographic profile is reviewed, and an analysis of push-pull factors influencing the decision to leave the home country finalises the theoretical part of the paper. The research model and method are presented in the methodology section. The results of the quantitative research of Lithuanian expat-preneurs in 24 countries are provided later. Discussion, conclusion, limitation, future research directions, and practical implications finalize the paper.

## Theoretical Background

### Theoretical Concepts

#### Self-Initiated Expatriates

The concept of SIEs was first introduced by Suutari and Brewster (2000), where the authors presented self-initiated expatriates in contrast with assigned expatriates, these being expatriates sent abroad by their employer (Arp et al., 2013). In comparison with AEs, SIEs are described as individuals who decide to look for international work-experience on their own initiative (Fitzgerald and Howe-Walsh, 2008; Andresen et al., 2014; Meuer et al., 2019; Andresen et al., 2020). In other words, they are conceptualised as free agents who cross organisational and national borders, unobstructed by barriers that constrain their career choices (Inkson et al., 1997).

Froese and Peltokorpi (2013) and Fee and Gray (2020) highlight that the demand for SIEs is on the rise, especially in Europe and Asia (McNulty et al., 2013). In addition, skilled SIEs constitute a valuable asset to the worldwide economy (Doherty and Dickmann, 2008; Fairlie, 2010; Hussain et al., 2019). Comparing statistical data of SIEs, 15 percent of them found a job on their own, 13 percent were sent by an employer, and 6 percent were recruited by a local company (Statistics Lithuania, 2016).

An essential characteristic of SIEs is that they leave their home country voluntarily for a predetermined period of time without the intention of becoming permanent citizens of the host country (Baruch et al., 2007; Al Ariss, 2010; Tharenou, 2010; Du Plessis, 2015; Vance and Paik, 2015; McNulty and Brewster, 2016; Meuer et al., 2019; Andresen et al., 2020). However, Al Ariss and Özbilgin (2010, p. 276) note that “the difference between SI expatriates and immigrant workers often remains implicit <…>. Both forms of expatriation are, in fact, not so different; many SI expatriates stay on a permanent basis and thus become permanent immigrants”. Therefore, another feature presenting the difference between migrants and expatriates is status in the host country. While foreigners do not always have a permanent permit or visa pass to stay in the host country, they remain as expatriates and after this their status changes to migrants (Al Ariss and Özbilgin, 2010; McNulty and Brewster, 2016). Any intention of becoming permanent citizens increases with the duration of the stay in the host country (Kumpikaitė-Valiūnienė and Žičkutė, 2017).

#### Pre-departure and Transitioned Expat-Preneurs

‘Expat-preneurs’ is a concept presented by Vance et al. (2016). It defines employees who go or remain abroad to start a new business in a host country, or who join in local host-country entrepreneurial activities (Vance et al., 2016). Therefore, we could describe expat-preneurs as self-employed expatriates.

Literature on the subject establishes three main differences between ethnic entrepreneurs and expat-preneurs (Vance et al., 2016; Girling and Bamwenda, 2018). Firstly, expat-preneurs stay temporarily in the host country, but ethnic entrepreneurs stay long-term. Also, expat-preneurs are not “necessity-entrepreneurs.” Finally, expat-preneurs usually come from a developed economy. It means expat-preneurs are in a more advantageous position than ethnic entrepreneurs, and they are not compelled by circumstances to stay in the host country or start their own business, but they do so of their own free will.

Vance et al. (2016) distinguish two different types of expat-preneurs. Some move abroad with an entrepreneurial purpose, or they try to expand their business from their home country to a new location. It means that these people have ‘entrepreneurial intentions’ before moving abroad, which explains individual willingness to start a business (Díaz-García and Jiménez-Moreno, 2010; Bastian, 2017). These expatriates are called ‘pre-departure expat-preneurs’ (Vance et al., 2016).

The other type of expat-preneurs do not have any intention of being self-employed before departure. They decide to move abroad, leaving their employer or the status of unemployment. After being in the host country for some time, they then start up their own business. This group of expatriates is called ‘transitioned expat-preneurs’ (Vance et al., 2016). In addition, Block and Wagner (2010) call such type of entrepreneurs ‘opportunity entrepreneurs’ as they are more likely to be alert to business opportunities than others.

The rising field of research on ‘pre-departure’ and ‘transitioned’ expat-preneurs and the need for empirical evidence provides the drive for further exploration of these types of expat-preneurs, and to identifying their characteristics and differences.

### Reasons of Foreigners to Become Entrepreneurs

Schumpeter’s theory addresses how entrepreneurs take risks in the pursuit of their goals and profits (Girling and Bamwenda, 2018). According to Kirkwood (2009), research on entrepreneurship motivation shows that both push and pull factors play a role for any individual entrepreneurs wanting to open a business. Patil and Deshpande (2019), when analysing female entrepreneurial motivation, note that among the pull factors are passion, independence, capital availability, and self-growth of a person, and among the push factors are economic necessity, financial burden, and loss of employment. In addition, environmental conditions for establishing and developing a business are important too.

Regarding foreigners, more factors need to be considered. Theoretical approaches that accommodate this emerging trend come from studies into international ethnic entrepreneurship and migration flows (Ilhan-Nas et al., 2011; Kumpikaitė-Valiūnienė and Žičkutė, 2017; Girling and Bamwenda, 2018). In addition, in the context of entrepreneurial venture, theories such as the cultural approach and the mixed embeddedness theory pointing out demographic and cultural traits (that a population shares) could explain the level of entrepreneurial success for foreigners (Masurel et al., 2002; Girling and Bamwenda, 2018; Arseneault, 2020).

The literature on migrant entrepreneurs focuses on migrants coming from undeveloped or developing countries to developed countries. The study by Moremong-Nganunu et al. (2018) on the biggest migrant entrepreneurial ethnic groups, such as Arabian, African, Asian, and South Asian, noted that entrepreneurial capabilities vary among different ethnic groups. Corresponding to the embeddedness theory, Bloch and McKay (2015); Rogerson and Mushawemhuka (2015), and Dannecker and Cakir (2016) found that good support in the host country and social-cultural capital are very important for entrepreneurial success. After literature analysis on migrant entrepreneurs, Agoh and Kumpikaite-Valiuniene (2018) highlighted the main conditions leading migrants to become entrepreneurs. These conditions include lack of jobs abroad, highly competitive job markets, lack of skills in certain cases, lack of language skills, cultural differences, discrimination in workplaces, determination to grow, personal entrepreneurial spirit, knowledge of the business, and internet business skills. Therefore, quite often the decision of migrant entrepreneurs to start their own business is based on necessity.

However, according to the expat-preneurial definition by Vance et al. (2016) expat-preneurs move from developed to developed countries. Therefore, we suppose that they should be less necessity-driven entrepreneurs. Usually, these expatriates are educated, and do not face any issues with language or discrimination. Factors that are important for them in starting their own business include a lack of career possibilities, a wish for independence and self-development, and finding a suitable business environment. We propose that some differences in pre-departure and transitioned expat-entrepreneurs might be revealed by looking at gender, age, and educational background.

### The Demographic Characteristics of Expat-Preneurs

Concerning the gender issue, until the 20th century, men predominated in moving to another country in order to pursue business opportunities. The scientific literature reflected this reality. Based on liberal feminist theory, men and women are essentially similar (Harding, 1987) and are seen as equally able to think rationally. Therefore, males and females and any subordination of females is connected with discrimination or structural barriers, such as unequal access to education. Bruni et al. (2004) noted three main barriers against female entrepreneurship. The first one could be described as the socio-cultural status of women, which is connected to the role of women with respect to responsibilities toward family, children, and housing. The second barrier is associated with the access to networks of information and assistance. Finally, the third highlighted barrier is access to capital. Women face problems searching for financial support and this is associated with a stereotype that ‘women can’t handle money’ and is connected to the two previous barriers. This corresponds with the mixed embeddedness theory (Girling and Bamwenda, 2018). Empirical evidence from the study of Azmat and Fujimoto (2016) on Indian female entrepreneurs in Australia highlighted that their success massively depended on their family embeddedness and cultural heritage.

According to the Global Entrepreneurship Monitor (2015), the phenomenon of entrepreneurship is growing among women, although they are still less involved in entrepreneurial activities in comparison to men. This can be seen in both developed and developing countries (Patil and Deshpande, 2019). Figures taken in 2014 for Lithuania show that 59,700 (8.9 percent) of females and 83,300 (12.9 percent) of males were self-employed. In 2015, the number for women slightly increased but the percentage slightly decreased: 58, 600 (8.6 percent), with both figures for men decreasing 59,900 (9.3 percent) (Department of Statistics, 2017).

Concerning entrepreneurial age and gender, studies by Brockhaus (1982) and Hisrich and Peters (1996) demonstrated that entrepreneurial decisions in general are taken between the ages of 25 and 40. However, some differences in relation to females could be noted. Langowitz and Minniti (2007) highlighted the most entrepreneurially active age of females was between 25 and 34 years, declining thereafter, which corresponds with the findings of Hisrich and Peters (1996). However, Still and Guerin’s (1987) earlier findings showed female entrepreneurs tended to be older - between the ages of 30 and 40. Also, Boden and Nucci (2000) analysed new business ventures with data on men and women from 1982 to 1987. This study pointed out differences in education and the amount of work experience, confirming a certain disadvantage in the case of female entrepreneurs. In addition, in the study by Gathenya et al. (2011) carried out in Kenya, the majority of female entrepreneurs were between 22 and 48 years. As Gathenya et al. (2011) highlight, this “age bracket is considered as the most entrepreneurially active age which contributes positively to the performance of enterprises.”

However, if speaking about the situation of expatriates, the situation is a bit different. A study on expatriates by Selmer et al. (2018) showed that expat-preneurs were older than company-employed expats with an average age of 44. Speaking about the level of attainment of entrepreneurs, Brockhaus (1982) noted that managers tend to be more highly skilled than entrepreneurs, but entrepreneurs tend to have a higher level of education than the general public.

Moreover, Leonard (2010) noted that entrepreneurship is popular among SIEs and particularly for women who usually are less involved in assigned expatriation agreements. The motivation for the expatriation and careers of female SIEs are complex and varied (Muir et al., 2014). Based on the study by Vance and McNulty (2014), 34 percent of females were SIEs and self-employed as consultants or small business owners versus 25 percent for men.

With this in mind, the assumption is that expat-preneurs could be older than regular entrepreneurs and, moreover, pre-departure expat-preneurs are older too as they had their own business in their home country already formulated. In comparison to men, more females are taking expat-preneur experience. However, there is not much evidence about the demographic characteristics of expat-preneurs, especially with regard to pre-departure and transitioned expatriates. Therefore, we propose the following hypothesis H1, in relation to demographic characteristics:

H1. There are significant differences between demographic characteristics of pre-departure and transitioned expat-preneurs.

### Push and Pull Factors Explaining Decision to Expatriate

The Push and Pull theory is the most popular theory explaining the process of human migration. Therefore, in order to analyse the reasons for the expatriation of pre-departure and transitioned SI expat-preneurs, push-pull factors were taken as the basis. In this sense, Kumpikaitė-Valiūnienė and Žičkutė (2017) reviewed the decision-making theories of migration and highlighted the main push-pull factors (see Table 1).

**TABLE 1:** Highlighted push-pull factors.

Push factors	**ECONOMIC**
	• Too low wages in a home country
	• Wage differences and income inequality
	• Low level of country’s economic development
	• Price politics of products
	• Person’s unemployment
	• Too few employment opportunities in a country
	• Not enough new workplaces in a country
	• Non-supportive tax system
	**NON-ECONOMIC/SOCIAL**
	• Personal life conditions
	• Study and education system
	• Not enough cultural centres, such as museums
	• Social conditions
	• The level of health care
	• Environmental conditions
	• Family reasons
	• Political corruption
	• Intolerance of personal attitudes/discrimination
	• Intention to spread your culture and religion
	• Wish for changes
Pull factors	**ECONOMIC**
	• Better opportunities to get a job
	• Lower cost of living
	• Higher income
	• Lower taxes
	**NON-ECONOMIC/SOCIAL**
	• A large number of home citizens in host country
	• Relatives living in this host country
	• The distance from the homeland
	• Language
	• Possibility for self-development
	• Political stability
	• More attractive weather
	• Better conditions of health care
	• Higher tolerance
	• The country’s prestige
	• Higher possibility for self-realisation

*According to Kumpikaitė-Valiūnienė and Žičkutė (2017).*

Economic or non-economic determinants can be attributed to “demand-pull” in the destination country, “supply-push” in the homeland, and network factors as the linkage between these two (Kumpikaitė-Valiūnienė and Žičkutė, 2017; Mihi-Ramirez et al., 2017). In conjunction with the SIE concept and the traditional migration theories, push and pull factors in the context of expatriation were applied.

Looking at the rationality that pre-departure and transitioned expat-preneurs moved abroad with different previous entrepreneurship experiences and, therefore, different primary intentions, we suppose their decisions to expatriate differ and so we propose the hypothesis H2.

H2: There are significant differences on push and pull factors between pre-departure and transitioned expat-preneurs.

**FIGURE 1:**
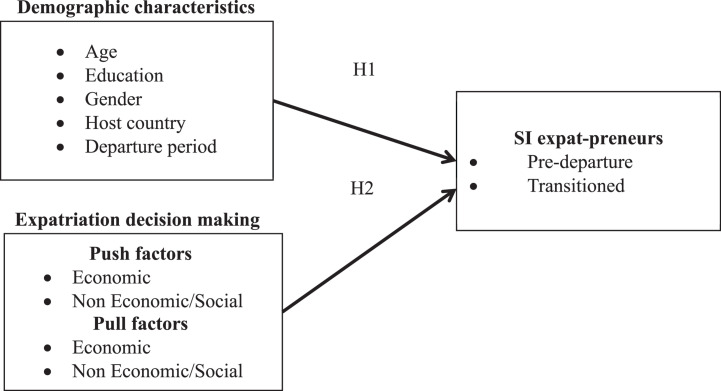
Research model.

To summarize, a theoretical model of study is presented in Figure 1.

## Methodology

### Context of the Research

Lithuania is a small EU country situated along the south eastern shore of the Baltic Sea, to the east of Sweden and Denmark. Its population is just 2.7 million, which has steadily decreased because of low birth rate and high expatriation. This decline started back in 1990 when Lithuania’s independence was restored after 50 years of Soviet occupation. The whole period after independence can be divided into four emigration waves (Kumpikaitė-Valiūnienė, 2019). The first wave includes the period of independence from1990 to 2003, the second wave started after joining the EU in 2004, the third wave started in 2009 with the economic crisis and Lithuania joining the Schengen Area, and the last wave started after joining the Euro zone in 2015. Most Lithuanians moved to more developed European countries and to the United States. Historically, Lithuanians used to migrate to the United States, with large numbers doing so from the end of the 19th century, and it remained the most attractive country to move abroad to until 2004 when Lithuania joined the EU. At this time, the United Kingdom, Ireland, Germany, and Spain became more popular and later, after the economic crises, Norway joined the list of favourite countries.

Although Lithuania is a developed country, it is economically weaker than the majority of older EU member states. Comparing information about the purchase power standard (PPS) and the average salary among EU countries, in 2015 the EU PPS average was 1.0, in the United Kingdom 1.7, Germany 1.6, Ireland 1.4, Spain 0.9, and in Lithuania 0.6 (Statistical office of the European Union Eurostat, 2016). At similar or lower levels were Slovakia, Latvia, Hungary, Czechia, Romania, and Bulgaria. Average salaries in 2014 were 2,690 EUR in Sweden, 2,597 EUR in the United Kingdom, 2,160 EUR in Ireland, 2,054 EUR in Germany, and 524 EUR in Lithuania (Fischer, 2018). In Lithuania, more than 80 percent of all companies are small and have up to only nine employees (Versli Lietuva, 2017). Therefore, career perspectives are very limited in Lithuania. In summary, Lithuanians move to foreign countries for better work, career, and economic perspectives and therefore provides a good example to analyse its expat-preneurs.

### Sample and Procedure

The survey method was selected for the research. Data gathering was completed online for several reasons: Shaffer et al. (2006) note that the response rate for expatriates is low, averaging 15 percent. In addition, it is difficult to access expat-preneur information as there is no available statistical data about Lithuanian expat-preneurs. Therefore, a decision was taken to separate expat-preneurs from the general group of expatriates.

An invitation to participate in the survey with a link to an online questionnaire was delivered to Lithuanian expatriates abroad through social media and websites. A call to participate in the study was also listed in Lithuanian expatriates’ webpages in different countries. The data was collected in October 2015 and from October to December 2016. Of course, the verification of the answers and their analysis also took much more time. In total, 1,586 respondents completed the questionnaire in October 2015 and 3,946 respondents participated in the survey from October to December 2016. Of the total participants, 308 respondents according to their current occupation were selected as the sample for this study. The sample was taken only from those respondents who had their own business outside of the home country, i.e., SI expat-preneurs. The status of SI expatriation was checked with the question ‘Who initiated your expatriation?’ and with a selection of multiple answers. In addition, all respondents did not have citizenship in the host country and, therefore, based on the approach we apply in this paper taken from Al Ariss and Özbilgin (2010) and McNulty and Brewster (2016), they could not be called migrants.

The sample consisted of two particular groups: pre-departure expat-preneurs and transitioned SI expat-preneurs. Of this, a total of 250 respondents (81.2 percent of the sample) started their businesses abroad with previous experience of being employed by others, studying, or being unemployed in Lithuania. These were attributed as being transitioned expat-preneurs. The remaining 58 respondents (18.8 percent of the sample) were self-employed entrepreneurs in Lithuania before leaving and represented pre-departure expat-preneurs in the sample. The demographic characteristics of pre-departure and transitioned expat-preneurs in the sample are presented in Table 2.

**TABLE 2:** Demographic characteristics of the sample.

		Pre-departure expat-preneurs	Transitioned expat-preneurs	Total sample
Respondents (*N*)		58	250	308
Gender (female: *N*, %)		30 (51.7)	179 (71.6)	209 (67.9)
Education (higher: *N*, %)		33 (56.9)	179 (71.6)	212 (68.8)
Age (*N*, %)	19 and less	0 (0.0)	1 (0.4)	1 (0.3)
	20–29	11 (19.0)	54 (21.7)	65 (21.2)
	30–39	10 (17.2)	133 (53.4)	143 (46.6)
	40–49	29 (50.0)	44 (17.7)	73 (23.8)
	50 and more	8 (13.8)	17 (6.8)	25 (8.1)
Host countries (*N*, %)	United Kingdom	11 (19.0)	61 (24.4)	72 (23.4)
	Norway	8 (13.8)	43 (17.2)	51 (16.6)
	United States	8 (13.8)	40 (16.0)	48 (15.6)
	Sweden	7 (12.1)	17 (6.8)	24 (7.8)
	Spain	7 (12.1)	11 (4.4)	18 (5.8)
	Ireland	4 (6.9)	12 (4.8)	16 (5.2)
	Germany	4 (6.9)	10 (4.0)	14 (4.5)
	Denmark	2 (3.4)	11 (4.4)	13 (4.2)
	*Others	7 (12.1)	45 (18.0)	52 (16.9)

* 15 other countries (Belgium, Holland, Iceland, Australia, Switzerland, Austria, Italy, Canada, New Zealand, Greece, France, Cyprus, Finland, Mexico, and Ukraine) with less than 2 percent (total sample) of respondents each.

In general, expat-preneurs from 24 countries participated in this study. The most attractive destination countries for the sample participants were the same as for the total Lithuanian population of expatriates, i.e., the United Kingdom, Norway, and the United States. Almost half of the respondents (46.4 percent) were 30–39 years old, with two additional groups having similar percentages: 40–49 years and 20–29 years old (respectively, 23.7 and 21.1 percent). Additionally, 67.9 percent of the sample were females (209 respondents), and 68.8 percent of the sample had a degree of higher education (212 respondents).

Respondents were divided into four groups based on the period of their departure. This grouping was done according to the four emigration waves in Lithuania, highlighted by Kumpikaitė-Valiūnienė (2019).

### Measures

The study had an exploratory nature with single question items for several key concepts and their constructs (Wanous et al., 1997). Push and pull factors of an economic and non-economic nature (respectively, 8 and 4 of push, 11 each of pull) were measured as independent variables for pre-departure or transitioned SI expat-preneurs’ paths. The list of factors provided and tested by Kumpikaitė-Valiūnienė and Žičkutė (2017) were used in the questionnaire. A general question about the reasons for initiating self-expatriation was given to respondents, along with the list of factors, unlimited choices, and including an open answer to provide any other factors not in the list that might come out of the expat-preneur’s experience. Each factor was coded as a separate variable (0 = not selected, 1 = selected).

The occupation of respondents was measured by two questions, asking for identification of the last occupation in their home country and the current occupation in their host country. The same list of 14 occupations (army officers, managers, specialists, technicians and younger specialists, office employees, services’ employees and sellers, qualified specialists of agriculture, qualified workers and masters, plant and machine operators and assemblers, unskilled workers, self-employed, students, unemployed, and housewives) was used for both questions with one open answer for other options, taken from Kumpikaitė-Valiūnienė and Žičkutė (2017). This measurement allowed for the selection of expat-preneurs only, composing the sample of 308 respondents, and affiliated them into a particular group of pre-departure or transitioned. A dummy variable for groups of pre-departure (1) and transitioned (0) expat-preneurs was created. In addition to demographic characteristics, such as gender, age, and education, another two characteristics related to Lithuania as the research context, such as the departure period and host country of respondents, were included. The departure period reflects the four Lithuanian migration waves (Kumpikaitė-Valiūnienė, 2019) and was measured by a question with five ranges for an answer (from 1 = until 1990, to 5 = since 2015 and later). The list of countries was provided for the host country, used for analysis as a nominal variable. Other demographic characteristics of respondents, like their gender, age, or education, were measured by a single question each. Age was recorded in five ranges (from 1 = 19 years and less, to 5 = 50 years and more) and used for further analysis. Education was measured in several levels and coded later into dummy variables (1 = secondary and professional, 2 = higher education).

### Methods of Analysis

A comparison of pre-departure and transitioned expat-preneurs’ demographic characteristics and push-pull factors was conducted using the Mann–Whitney *U* rank test. Logistic regression was used for measuring the impact of push and pull factors (independent variables), departure period and host country (control variables from the research context), and demographic characteristics like gender, age, and education (control variables) on pre-departure or transitioned SI expat-preneurs’ paths (dependent variable).

### Results

#### Comparison Analysis

Two independent groups of pre-departure and transitioned expat-preneurs were analysed according to demographic characteristics and push and pull factors of expatriation. Differences between the two groups were found in cases of gender, age, and education but not in the departure period (see Table 3), confirming the Hypothesis 1 (H1).

**TABLE 3:** Comparative analysis matrix for demographic variables.

	*M*	*SD*	Mean rank		Mann–Whitney *U*	*Z*
			Transitioned expat-preneurs	Pre-departure expat-preneurs		
Expat-preneurs (0 = transitioned)	0.19	0.39				
Gender (1 = male)	1.68	0.47	160.26	129.66	5809	−2.915**
Education (1 = secondary and professional)	1.69	0.46	158.76	136.12	6184	−2.175*
Age (1 = 19 and less)	3.18	0.87	144.70	193.93	4905	−4.066**
Departure period (1 = until 1990)	3.33	0.95	144.73	156.20	6281.5	−0.977

* p < 0.05, ** p < 0.01.

Comparative analysis results show that pre-departure expat-preneurs were older and less educated than transitioned expat-preneurs, and there were more males than females among them. Looking at the work positions, 15.8 percent of transitioned expat-preneurs worked in the services sector, 14.5 percent studied, and 11.2 percent were specialists in Lithuania before they expatriated. The biggest amount (more than 40 percent) within both groups left Lithuania during the third emigration wave. Of the pre-departure expat-preneurs, 90.7 percent were satisfied with their career, compared to 80.5 percent of transitioned expat-preneurs.

The analysis of all push and pull factors for expat-preneurs’ groups (pre-departure and transitioned expat-preneurs) revealed significant differences only for six single factors (see Table 4).

**TABLE 4:** Comparative analysis matrix for expatriation factors.

Expatriation factor	*M*	*SD*	Mean rank		Mann–Whitney *U*	*Z*
			Transitioned expat-preneurs	Pre-departure expat-preneurs		
Non-supportive tax system	0.29	0.45	147.46	184.84	5490	−3.681**
Too few employment opportunities	0.15	0.36	158.10	138.97	6349	−2.367**
Political corruption in Lithuania	0.32	0.47	145.66	192.62	5039	−4.473**
Family reasons	0.22	0.41	157.96	139.59	6385	−1.981*
Higher possibility for self-realisation	0.45	0.50	149.68	175.28	6045	−2.288*
Prestige of host country	0.11	0.32	151.78	166.21	6571	−2.021*

* p < 0.05, ** p < 0.01.

We found differences in these economic push factors between expat-preneurs (pre-departure and transitioned). Our results show that a significant pushing effect from expat-preneurs is a non-supportive tax system. This was more important for pre-departure expat-preneurs than for transitioned expat-preneurs. However, having too few employment opportunities was a more important push factor for transitioned expat-preneurs. Similar effects were found in non-economic push factors. Political corruption in Lithuania was a more common non-economic push factor for pre-departure expat-preneurs, while family reasons played a more important role for transitioned expat-preneurs.

Only two non-economic pull factors from the whole group revealed differences between pre-departure and transitioned expat-preneurs, with differences being of the same direction. The higher possibility of self-realisation, as well as host country prestige, revealed a stronger pull effect to pre-departure expat-preneurs than to transitioned ones.

**FIGURE 2:**
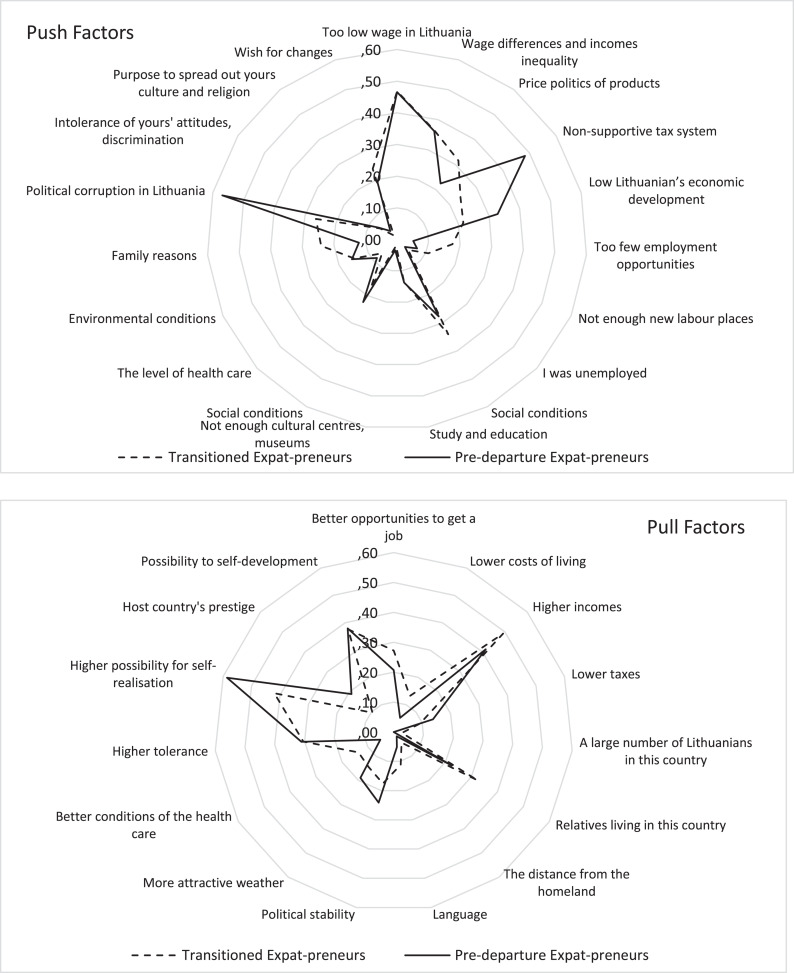
Profiles of pre-departure and transitioned expat-preneurs according to expatriation factors.

Comparing results in the profiles of pre-departure and transitioned expat-preneurs (see Figure 2), differences existed, but in general, they appeared only in the case of six factors from 34, so it confirmed Hypothesis H2, but just for these factors.

#### Regression Analysis

According to the theoretical model, three models were tested using logistic regression (see Table 5). The results showed that push and pull factors (model 1) that differ between pre-departure and transitioned expat-preneurs correctly predicted 81.8 percent of the expat-preneurs’ type. Adding demographic variables to the models (model 2 and model 3) raised the prediction up to 86.3 percent with an R square of 0.375.

**TABLE 5:** Results of logistic regression.

	Model 1			Model 2			Model 3		
	β	*SE*	Wald	β	*SE*	Wald	β	*SE*	Wald
Constant	–2.093	0.287	53.297**	–1.637	0.597	7.529**	1.009	1.150	0.770
**Expatriation (independent) variables**									
Non-supportive tax system	0.871	0.351	6.151*	0.864	0.357	5.851*	0.392	0.419	0.878
Too low employment opportunities	–1.954	0.655	8.896**	–2.044	0.662	9.541**	–2.083	0.740	7.925**
Political corruption in Lithuania	0.873	0.353	6.098*	0.799	0.358	4.985	0.668	0.420	2.533
Family reasons	–0.420	0.459	0.839	–0.477	0.465	1.052	–0.635	0.524	1.470
Prestige of host country	0.187	0.440	0.180	0.076	0.455	0.028	0.156	0.505	0.096
Higher possibility for self-realisation	0.561	0.329	2.897	0.603	0.339	3.174	0.687	0.380	3.265
**Demographic (control) variables**									
Departure period (L)						3.489			4.817
				–18.885	27957.878	0.000	–19.988	27770.198	0.000
				–0.057	0.621	0.008	–1.199	0.703	2.906
				–0.870	0.648	1.803	–1.312	0.720	3.324
				–0.114	0.569	0.040	–0.499	0.587	0.722
Host country				–0.018	0.025	0.494	–0.045	0.030	2.193
Gender							–0.531	0.393	1.822
Age(L)									27.532**
Age(1)							–20.864	40192.970	0.000
Age(2)							–1.904	0.727	6.853**
Age(3)							–2.290	0.644	12.630**
Age(4)							0.072	0.589	0.015
Education							0.889	0.407	4.777*
*Nagelkerke R^2^*	0.192			0.213			0.375		

L, Last category is used as an indicator, i.e., departure period 5, Age 5.

* p < 0.05, ** p < 0.01.

In all three models, too low employment played an important economic push role on the path of pre-departure and transitioned expat-preneurs. In the first and second models the additional impact of a non-supportive tax system can be seen. The first model also included the impact of political corruption in Lithuania. Hereafter, age and education were significant in the third model, but not gender, improving the R square even more. In summary, all three models represented a good fit and confirmed the impact of tested variables on the types of expat-preneurs.

## Discussion

Traditionally, most theories and studies describe foreign entrepreneurs as people who migrate to more developed countries out of necessity. Our results highlight how entrepreneurs from developed countries deepen their motivations, and the differences between pre-departure and transitional expat-preneurs, through a focus on expatriation reasons and demographic characteristics.

Theories about international entrepreneurship, such as the cultural approach and the mixed embeddedness theory, have had a limited empirical evidence so far. Our results support them confirming the relevance of a demographic profile for different types of expat-preneurs. Thus, the analysis of international business activity should include differences between traditional ethnic migrants and new expatriate pre-departure and transitioned entrepreneurs, broadening the scope of the analysis of such theories.

In this line, our results highlight the existence of discrepancies between international ethnic entrepreneurs (South to North) and expat-entrepreneurs (from developed countries), thus contributing to research calling for space to include expat-preneurs in entrepreneurship theories (Andresen et al., 2014, 2020; Vance et al., 2016; Girling and Bamwenda, 2018; Meuer et al., 2019). Some new insights about gender issues were revealed in the study.

The gender issue matters when studying global entrepreneurs. Thus, any overseas venture is a process experienced differently by males and females and therefore could be considered to be sex-selective. Males especially dominate among assigned expatriates. Tendencies have been changing in the last 20 years, and the gender approach in international entrepreneurship processes has become very important. Besides this fact, the data analysis of this study found that more females who were not self-employed in Lithuania became expat-preneurs in their host countries. This could be explained by the fact that more females left their home country due to family reasons and therefore came to entrepreneurial activities later (Leonard, 2010). Our study revealed that more females are transitioned expat-preneurs. It is probable that after some time spent abroad, females see expat-preneurship as an opportunity to be employed (Lewin, 1998) and/or to take up and follow activities that they have always wanted to do.

No statistical inference was found in education according to gender in our sample. This did not correspond with the findings of Boden and Nucci (2000), who highlight that females are seen as having insufficient education or experience. Such findings provided new insights into expat-preneurs that, based on their nature, they are less necessity-driven entrepreneurs than migrant entrepreneurs are. Expat-preneurs come from developed countries and their education does not depend on gender, and the majority of them have reached a level of higher education. However, these results based on a one country case provided only a few insights and they need deeper analysis and comparison with other developed and developing countries.

Looking at other demographic characteristics, results show that pre-departure expat-preneurs are older and less educated than transitioned expat-preneurs. It partly corresponds with the study of Selmer et al. (2018), which showed that expat-preneurs were older than company-employed expatriates. According to the study, some respondents who graduated from high school abroad and decided to start their own business were younger and more highly educated.

As previously mentioned, the business environment is an important factor for entrepreneurship (Kirkwood, 2009; Patil and Deshpande, 2019). Due to the specifics of our study, analysis was based on expatriation push-pull factors and economic indicators of the home and the main host countries. Political corruption in the home country and a non-supportive tax system were identified as the most important expatriation factors for pre-departure expat-preneurs. This showed that people were looking for better business opportunities abroad. As an example, the 2015 corruption perception index (where 0 means highly corrupt and 100 very clean) was 81 in the United Kingdom, 76 in the United States, 89 in Sweden, 88 in Norway, 75 in Ireland, 81 in Germany, 91 in Denmark, and 58 in Spain in comparison to 59 in Lithuania (Transparency International, 2018). Based on this data, we saw that the main destinations for Lithuanians were less corrupt than Lithuania. It was more complicated to compare tax systems in different countries as they depend on types, size of business, and various regulations in each country. In terms of corporate tax in these destination countries, this varied from the lowest of 12.5 percent in Ireland, up to 40 percent in the United States, with Lithuania having 15 percent (KPMG, 2018). Comparing the ranking of 80 countries in 2019 in terms of where best to start a business, Lithuania was #53, the United States #11, the United Kingdom #13, Sweden #18, Germany #25, and Spain #33 (U.S. News, 2020). However, the business environment is even more important in order to be successful in starting a business. Forbes (2015) provided the list of Best Countries for Business by grading 144 nations on 11 different factors which encourage entrepreneurship [property rights, innovation, taxes, technology, corruption, freedom (personal, trade, and monetary), red tape, investor protection, and stock market performance]. According to these factors, Denmark was #1, Norway #3, Ireland #4, Sweden #5, United Kingdom #10, Germany #18, and the United States #22 in 2015. Summing up, based on reviewed factors and the conducted study, Lithuania’s general business environment was not very attractive and was the reason for pre-departure entrepreneurship.

The most important non-economic pull factors are a higher possibility for self-realisation and the possibility of self-development. This shows that the sample of analysed self-employed respondents truly represents expat-preneurs, as they left their country of origin for reasons connected with better job opportunities. This could be related to the classical Schumpeter Theory (Girling and Bamwenda, 2018), meaning that pre-departure expat-preneurs pursue better opportunities by establishing themselves in other countries, as does the traditional ethnic migrant. However, research by Stone and Stubbs (2007) on the motivations of 41 British expatriate entrepreneurs managing 71 family businesses in other countries, such as Spain and France, found that, rather than profit, they settled in those countries to improve their lifestyle. According to Schumpeter, all expat-entrepreneurs would have the advantage of possessing innovative and risk-taking skills that enable them to achieve success.

Our results allow us to qualify the assumptions of Schumpeter’ Theory and Stone and Stubbs (2007), so that in the case of pre-departure entrepreneurs, they would use their skills to take advantage of the best opportunities that exist in other countries, such as a more favourable tax system, less corruption, and better labour market conditions. But also, in the case of transitioned entrepreneurs (already established in the destination country and without the pressure of home country circumstances), entrepreneurship is motivated by improved lifestyle, greater prestige, and self-realisation.

### Implications for Managerial Practice

A deeper understanding of expat-preneur phenomena is useful for both the home and host countries. Received results could be useful for Lithuania, as policy makers should consider the main push factors behind moving business abroad, like political corruption and taxes and their burden. Possible solutions to prevent other entrepreneurs expatriating to other countries as well as how to motivate expat-preneurs to start transnational business and expanding it into home countries might be elaborated. This would help to bring financial and human capital into countries that lose valuable employees, such as Lithuania. In addition, countries in Central and Eastern Europe that experience similar flows and tendencies of expatriation might also benefit from the findings of this research.

In addition, according to Vance et al. (2016, p. 212), ‘expat-preneurs can further contribute to the long-term economic health and growth of a host country through knowledge transfer.’ They contribute not only knowledge and human capital, but also physical capital, and they pay taxes and contribute toward the development of the host country. According to human capital theory (Chorny et al., 2007), expatriates are young and qualified individuals and, in addition, our study revealed that transitioned expat-preneurs are younger that pre-departure ones. Therefore, the decision to move abroad is an investment because an individual increases his or her employment perspectives (Sjaastad, 1962). Not only countries, but also organisations in Lithuania and CEE countries, need to encourage changes in the areas that influence the factors of expatriation.

## Conclusion

It should be noted that expatriation is a growing phenomenon in developed countries. People expatriate to where they see better possibilities for employment, self-realisation, and personal development. Often, these expatriates become self-employed and turn into expat-preneurs. The Lithuanian case presented here, studying the similarities and differences of expat-preneurs, contributes to the exploration of the expatriation process and provides a profile of an expat-preneur. Introducing demographic characteristics helps to forecast the type of expat-preneur. Differences are found in the cases of gender, age, and education. Pre-departure expat-preneurs are older and less educated than transitioned ones. According to the results, more males are pre-departure expat-preneurs and more females are transitioned expat-preneurs.

There are more similarities than differences between the expatriation factors of pre-departure and transitioned expat-preneurs, bridging them more than dividing them. With regard to differences, the results show that pre-departure expat-preneurs are pushed to move abroad because of a better business environment, while they are pulled by the higher possibility of self-realisation as well as the prestige of the host country. At the same time, transitioned expat-preneurs are pushed more by family reasons, along with too few employment/career opportunities.

The present study contributes to the expatriation research field by empirically tested pre-departure and transitioned expat-preneur phenomenon based on their demographic characteristics and decision to leave their home country. Our results extend the scope of traditional theories of entrepreneurship, such as the cultural approach and the mixed embeddedness theory, as well as Schumpeter’s theory of the case of expat-preneurs.

### Limitations and Guidelines for Future Research

Due to difficulties in directly accessing expat-preneurs, and instead taking them as a sample from a general group of expatriates, not all the questions were connected with their entrepreneurial activities, but this is a very small number among a large number of questions which did not affect the purpose of the research. In addition to a quantitative nature of the research, the majority of respondents had not indicated what kind of business they were in. Therefore, we propose as a future research line to study the diversity and popularity of business types among Lithuanian expat-preneurs. Furthermore, respondents were from 24 different countries. Such a limited geographic spread did not allow an analysis in accordance with countries that might be valuable in exploring the impact of the host country on expatriation decision making. However, this also means some advantages in the study of their demographic characteristics, such as belonging to the same culture. In addition, as indicated in Section “Sample and Procedure,” focusing on a small country with high migration rates is convenient for our analysis of push-pull factors and migration. In any case, we would like to extend and replicate this research in the future by including a sample of more countries with similar characteristics, or groups of countries with differences between them.

Decisions to locate businesses in the host and/or home countries usually depend on different tax rates, growth prospects, laws, and attitudes toward foreign businesses (Vemuri, 2014). However, in this case, due to the shortage of time to access expat-preneurs, the push-pull factors were analysed as the reason to expatriate but not in the context of the decision to establish a business abroad. However, we propose as a future line of research the perspective of the destination country. In addition, the time when transitioned expat-preneurs started their business abroad after they moved to the host country was not controlled. Such data would contribute to the exploration of expatriates’ entrepreneurship field.

One of the main shortages was a lack of questions about marital status and children. Without this, it was not possible to complete an analysis of the family’s impact on the decision of respondents to move and to become entrepreneurs. Gender issues are already partly covered, but they are important in developing this research further as the majority of our expat-preneurs were females. In addition, the gender issue should be studied further in terms of ‘entrepreneurial intentions’ (Díaz-García and Jiménez-Moreno, 2010) and ‘accidental entrepreneur’ (Lewin, 1998) differences because females, as previously mentioned according to Bruni et al. (2004), face three main barriers in becoming entrepreneurs. Moreover, there is still a lack of studies into what extent pre-departure and transitioned expat-preneurship in their various forms are influenced by gender.

As for the motivations for expatriation, even taking into account the above limitations, it would be interesting to continue this research by delving into the similarities and differences between different ethnic expatriates, and also expand the sample to other nationalities. For example, corresponding to a cultural approach, Andrejuk (2017) in studying a unique case of EU-15 and the EU-12 entrepreneurs in Poland, revealed that cultural differences play an important role in entrepreneurial success. Also, entrepreneurs from the EU-12 succeeded in their business when they fully integrated into the host communities but expatriates from the United Kingdom and Spain were successful when they employed their cultural heritage. Therefore, more studies on ethnic expat-entrepreneurs would allow the scope of entrepreneurship theories to be extended.

## Data Availability Statement

The raw data supporting the conclusions of this article will be made available by the authors, without undue reservation.

## Ethics Statement

Written informed consent from the participants was not required to participate in this study in accordance with the national legislation and the institutional requirements. Ethical review and approval was not required for the study on human participants in accordance with the local legislation and institutional requirements.

## Author Contributions

VK-V, JD, and AMR contributed to the design and implementation of the research, to the analysis of the results, and to the writing of the manuscript. All authors contributed to the article and approved the submitted version.

## Conflict of Interest

The authors declare that the research was conducted in the absence of any commercial or financial relationships that could be construed as a potential conflict of interest.
